# Extracting

**Published:** 1907-10

**Authors:** R. T. Kennealy

**Affiliations:** Wexford, Ireland.


### EXTRACTING.

I note an article in the July issue of the Journal by Dr. Gustavus North, which I beg in my humble way to confirm. I can never understand any dentist having the interests of his clients at heart extracting any tooth that can possibly be saved. There is only one type of case in which I counsel extraction of a tooth—sound or carious—and that is where a single incisor or bicuspid remains and if left will interfere with the suction and the appearance of the denture to be inserted.

R. T. Kennealy, L. D. S., R. C. S. E.,

Wexford, Ireland.

